# Supplementary figures and images for: Electroencephalographic response to transient adaptation of vestibular perception
Source: J Physiol. 2022 Jul 7;600(15):3517–35. doi: 10.1113/JP282470 (PMC9544486; doi:10.1113/JP282470)

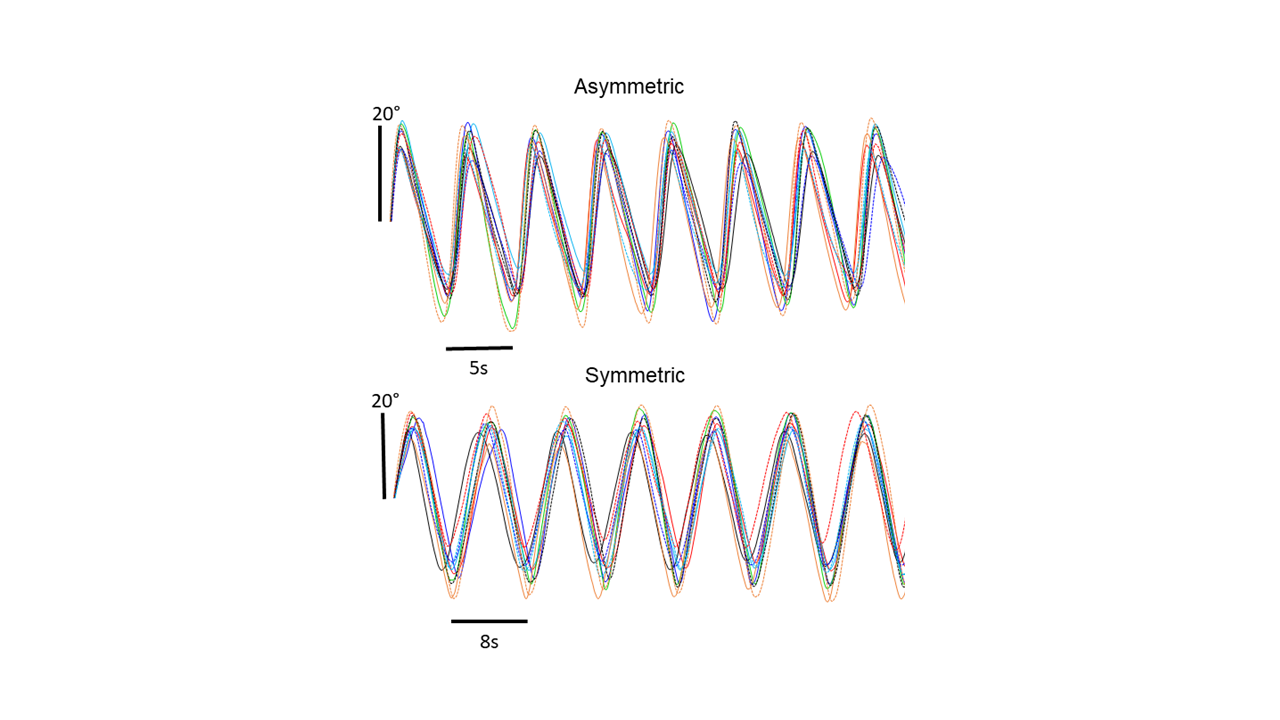

Supplement: Supplementary file 3 — Figure S1 [file TJP-600-3517-s004.png]

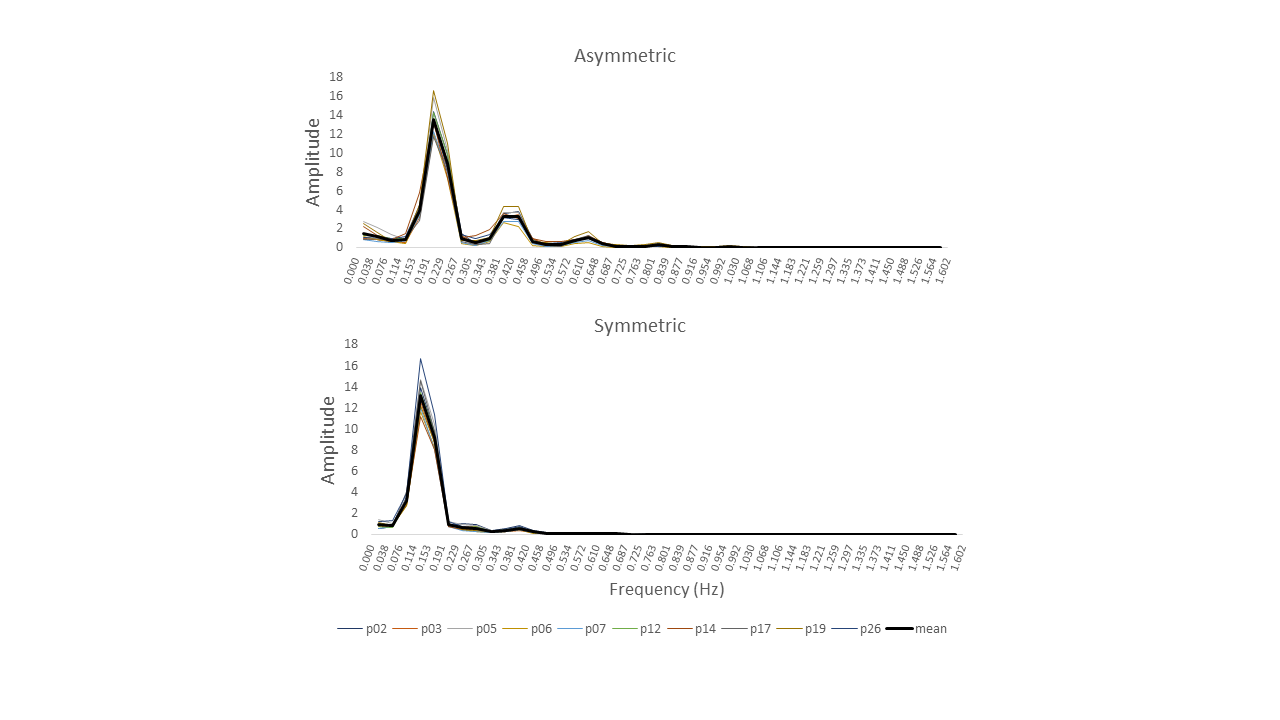

Supplement: Supplementary file 4 — Figure S2 [file TJP-600-3517-s003.png]
